# Emergence of SARS-CoV-2 resistance mutations in a patient who received anti-SARS-COV2 spike protein monoclonal antibodies: a case report

**DOI:** 10.1186/s12879-021-06902-1

**Published:** 2021-12-07

**Authors:** Honorine Fenaux, Romain Gueneau, Amal Chaghouri, Benoît Henry, Lina Mouna, Anne-Marie Roque-Afonso, Christelle Vauloup-Fellous

**Affiliations:** 1 AP-HP, Hôpital Paul Brousse, Service de Virologie, 94800 Villejuif, France; 2 Université Paris-Saclay, AP-HP, Hôpital Bicêtre, Service des maladies infectieuses, 94270 Le Kremlin Bicêtre, France; 3 Université Paris-Saclay, AP-HP, Hôpital Paul Brousse, Service de Virologie, INSERM U1193, 94800 Villejuif, France; 4 Laboratoire de Virologie, Hôpital Paul Brousse, 12 avenue Paul Vaillant Couturier, 94800 Villejuif, France

**Keywords:** SARS-CoV-2, Monoclonal antibodies, Resistance mutations selection, Case report

## Abstract

**Background:**

To manage severe or potentially severe cases of CoronaVirus Disease 2019 (COVID-19), therapeutic monoclonal antibodies targeting Spike protein of Severe Acute Respiratory Syndrome Coronavirus-2 (SARS-CoV-2) have been designed. It has been noted in vitro that upon exposure to these treatments, mutations could be selected.

**Case presentation:**

We here report the case of an immunosuppressed patient infected with a B.1.1.7 variant, who received a combination of monoclonal antibodies, and subsequently selected mutations K417N, E484K and Q493R on Spike protein of SARS-CoV-2.

**Conclusions:**

Our case raises the importance of monitoring SARS-CoV-2 mutations in patients receiving monoclonal antibodies and having persistent excretion of the virus, in order to offer optimal management of their infection, and strengthen prevention measures to avoid subsequent transmission of these selected variants.

## Background

CoronaVirus Disease 2019 (COVID-19) has emerged at the end of year 2019 and has rapidly spread throughout the planet. COVID-19 is due to the Severe Acute Respiratory Syndrome Coronavirus 2 (SARS-CoV-2). Although usually asymptomatic or mild, COVID-19 can be severe and sometimes fatal in specific populations, especially immunocompromised hosts. At the end of February 2021, monoclonal antibodies (mAbs) directed against the Spike (S) glycoprotein of SARS-CoV-2 have been approved in France, for the treatment of patients at high risk of severe COVID-19 [1, 2].

It has been assumed that using these antibodies might lead to the emergence of neutralization-resistant variants, but to date, this phenomenon has only been evidenced in vitro [3].

We report here the case of mutations of amino acids 417, 484 and 493 of the S protein selected in an immunocompromised patient receiving mAbs.

## Case presentation

**Fig. 1:**
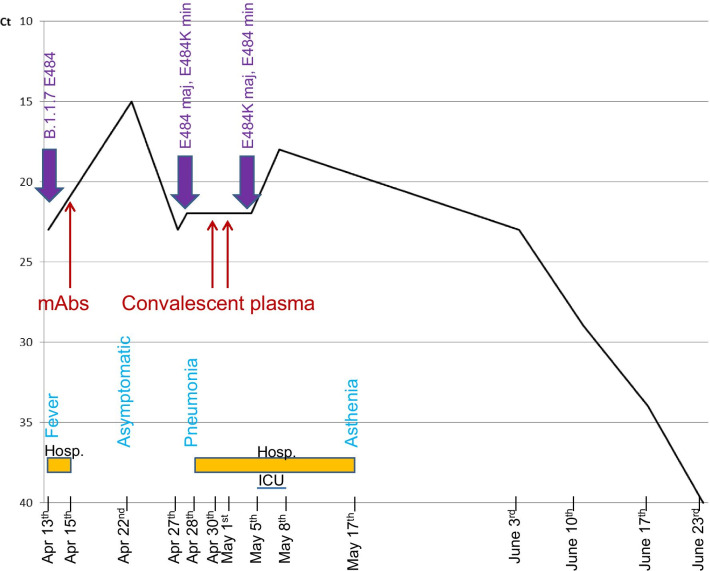
Evolution of SARS-CoV-2 PCR Ct during COVID-19 course. Mutations detected, treatment received, main clinical signs and hospitalization periods are reported. *Hosp* hospitalization, *ICU* intensive care unit, *maj* majority, min: minority

A 55-year-old man, with a past history of stage I follicular lymphoma, receiving obinutuzumab plus CHOP chemotherapy (cyclophosphamide, doxorubicine, vincristine, prednisone), was initially admitted to our infectious diseases department for the management of acute knee arthritis, finally considered to be reactive arthritis. While hospitalized, he developed fever and was diagnosed with COVID-19 on April 13th on a positive SARS-CoV-2 RT-PCR (22.1 and 24.8 Ct for E and N2 genes respectively, Xpert® Xpress SARS-CoV-2, Cepheid diagnosis, Sunnyvale, California, USA). Variant screening with TaqPath assay (ThermoFisher, Waltham, Massachusetts, USA) and VirSNiP SARS-CoV-2 Spike 484K-501Y assay (TIB Molbiol, Berlin, Germany) allowed the detection of a B.1.1.7 variant further confirmed by Sanger sequencing (69–70 and 144 deletions, and N501Y mutation on the S gene) with glutamic acid on position 484 (wild type) (Fig. 1). Initially, the patient did not present with any respiratory symptoms.

On April 15th, given the recent diagnosis of SARS-CoV-2 infection and the immunocompromised status (including hypogammaglobulinemia: total gammaglobulins 5.5 g/l), the patient received one dose of an association of two anti-S mAbs: Bamlanivimab 700 mg and Etesevimab 1400 mg [4] and was discharged home.

On April 22nd, 9 days after the first positive PCR, the patient was evaluated as an outpatient: he was afebrile, had no respiratory symptoms and had a normal oxygen saturation; however, SARS-CoV-2 RT-PCR (Alinity m, Abbott Diagnostics, Chicago, Illinois, USA) performed on a nasopharyngeal swab remained highly positive (Ct: 15.1) (Fig. 1).

**Fig. 2:**
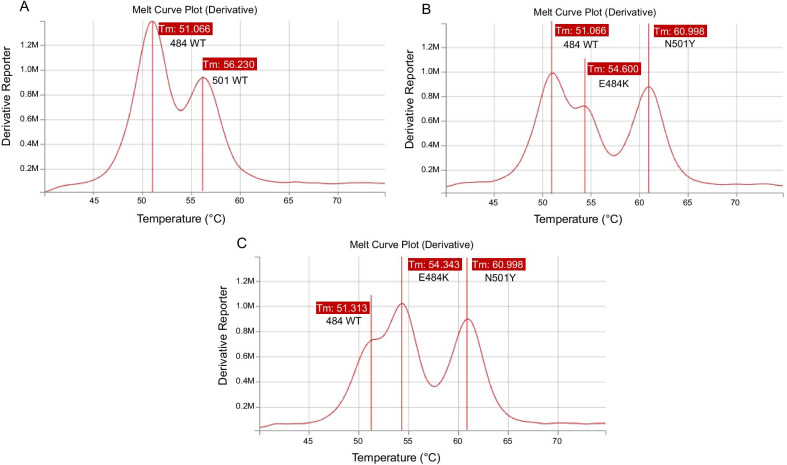
TIB Molbiol results, melt curve. **A** Positive control, wild type, one peak at 51 °C for wild type position 484, a second peak at 56 °C for wild type position 501. **B** Naso-pharyngeal swab collected on April 28th: a double peak is observed for position 484: the main first peak at 51 °C (wild type 484) and a second smaller one at 54.6 °C (E484K); the third peak at 61 °C is N501Y. **C** Bronchoalveolar lavage collected on May 5th where a double peak is again observed: the main peak at 54.3 °C (E484K) and a smaller one at 51.3 °C (wild type 484); the N501Y peak at 61 °C is unchanged. *WT* wild type, *Tm* melting temperature

**Fig. 3:**
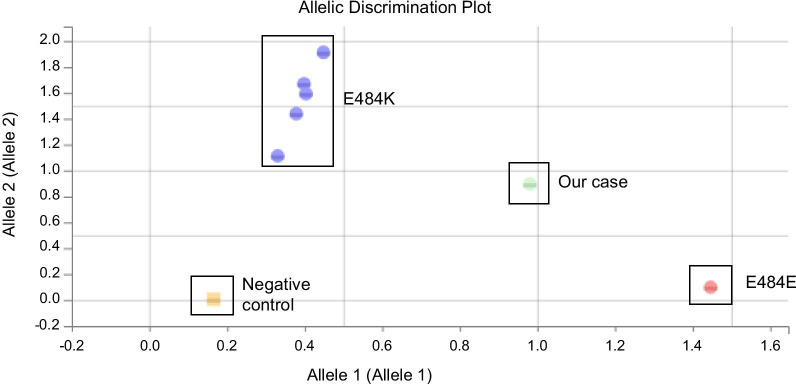
ThermoFisher VIRSNiP assay (ThermoFisher, Waltham, Massachusetts, USA) results. No amplification for the negative control; wild type samples (E484E) segregate on the low right; E484K samples segregate at the top; sample collected from our patient is observed in an intermediate location between wild type samples and E484K samples

On April 28th, the patient presented with respiratory symptoms and hypoxemia that prompted hospital admission. Repeat naso-pharyngeal swab remained positive for SARS-CoV-2 (RT-PCR, Alinity m, Ct: 21.7). Thoracic computed tomography ruled out pulmonary embolism and showed bilateral ground-glass opacities, highly suggestive of COVID-19. Given this clinical deterioration, convalescent plasma therapy infusion was planned, and a new screening for 484K mutation (484K-501Y TIB Molbiol) was performed, showing a double peak at position 484 (Fig. 2B). The first main peak had a melting temperature compatible with the wild type 484, and a second smaller peak had a melting temperature compatible with substitution E484K. The hypothesis of a double viral population was further suggested by the 484K VIRSNiP assay (ThermoFisher, Waltham, Massachusetts, USA), which gave an “intermediate” result (Fig. 3), compatible with the existence of a double viral population. To exclude any contamination, all investigations were performed twice on different aliquots of the same sample collected on April 28th. On April 30th and May 1st, while still presenting hypoxemic pneumonia, the patient finally received convalescent plasma therapy (Etablissement Français du Sang) containing high titres of anti-SARS-CoV-2 antibodies (Fig. 1).

On May 4th, worsening hypoxemia necessitated intensive care unit (ICU) admission, to receive high-flow oxygen therapy. On May 5th, ICU SARS-CoV-2 RT-PCR was still positive on a bronchoalveolar lavage with the Simplexa™ COVID-19 Direct assay (22.1 Ct for both S and ORF1a genes, Diasorin, Saluggia, Italy), and a double peak at position 484 was again observed with the 484K-501Y TIB Molbiol assay (Fig. 2C). This time, the highest peak had a melting temperature compatible with the E484K substitution, while the smaller one was compatible with the wild type 484.

On May 8th, the patient was discharged from the ICU, still receiving nasal oxygen therapy (2 L/min). SARS-CoV-2 RT-PCR was still positive on a naso-pharyngeal swab (18.3 Ct, Alinity m). On May 17th, the patient was discharged home, weaned from oxygen and presenting no general symptoms except persisting asthenia. On June 3rd and June 10th, the patient remained persistently positive for SARS-CoV-2 RT-PCR on naso-pharyngeal swabs (22.67 and 28.59 Ct respectively, Alinity m). On June 17th, the viral load was lower (33.75 Ct, Alinity m) and finally on June 23rd, SARS-CoV-2 RT-PCR was negative (Alinity m) (Fig. 1).

**Fig. 4:**
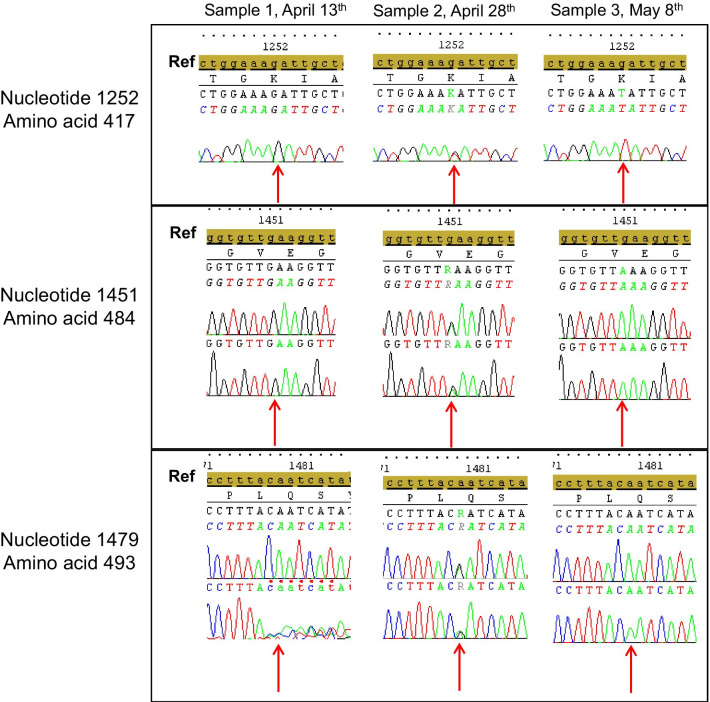
Sanger sequence of S protein on the three samples and focused on three different nucleotide/amino acid positions. Ref: reference sequence (20A.EU2), red arrow: nucleotide position of interest

Sanger sequencing of the S gene was performed on three samples and analysed on SeqScape 4 software, aligned on reference sequence 20A.EU2. It showed a typical B1.1.7 variant on the first sample (April 13th); double populations: K417K/N, E484E/K and Q493Q/R on the 2nd sample (28th April), and E484K and K417N on the 3rd sample (May 8th) (Fig. 4).

## Discussion and conclusion

We report here the case of a COVID-19 immunocompromised patient presenting protracted and elevated viral loads of SARS-CoV-2 (< 25 Ct regardless of the assay) in respiratory samples during 51 days despite mAbs therapy with Bamlanivimab and Etesevimab. Close examination of the virus with screening and sequencing assays highlighted the emergence of mutations at several positions targeted by mAbs (484, 417 and 493).

Since the beginning of the COVID-19 pandemic, several treatments have been evaluated and among those, mAbs showed encouraging results, especially in reducing respiratory viral load [4, 5]. Variants with an E484K substitution within S glycoprotein can escape acquired immunity, either through prior natural infection or vaccination, at least partially [6, 7]. Indeed many mAbs, convalescent sera and mRNA vaccine-induced immune sera show reduced inhibitory activity against SARS-CoV-2 harbouring this substitution in vitro [8, 9]. The risk of selecting a mutation, especially at position 484, has already been raised [3, 10]. Liu et al. have shown the emergence of this substitution under mAbs selective pressure in vitro [3]. Other mutations on the S protein liable to escape mAbs effect have been described, especially K417N and Q493R [11].

It remains to be determined if the probability of such emergence of mutations is linked to the immunocompromised status of the patient. mAbs can be administered in case of high risk of severe COVID-19, which includes immunosuppressed patients but also patients suffering from other conditions such as obesity, chronic respiratory failure, diabetes, cardiac failure, pulmonary fibrosis etc. [1]. In other viral infections such as Herpesviridae, emergence of resistance mutations does not result from long-term treatment alone, but mainly from the patient being immunosuppressed [12]. Administration of mAbs in high risk immunocompetent patients might not lead to selection of resistant mutants, whereas administration of mAbs in immunosuppressed patients might.

Despite promptly receiving anti-S protein mAbs after the SARS-CoV-2 infection diagnosis, our patient developed severe COVID-19, highlighting the therapeutic difficulties associated with COVID-19 in immunocompromised hosts. The severity of the disease in lymphoma patients receiving chemotherapy combined with anti-CD20 monoclonal antibodies has been reported [13, 14]. The delayed onset of symptomatic disease, compared to the general population, has also been reported in this specific population [15]. Clinical evolution was finally favourable under symptomatic measures and convalescent plasma therapy. Similarly, previous reports have emphasized the potential interest of convalescent plasma in lymphoma patients receiving anti-CD20 antibodies [16–19].

Finally, an important concern is also the opportunity for these “selected variants” to be transmitted to other individuals and its potential consequences. Patients having received mAbs but still excreting virus may be discharged upon an improving clinical condition and might contaminate their relatives, resulting in the circulation of the “selected variant”.

Our case emphasizes the importance of monitoring SARS-CoV-2 mutations in patients receiving mAbs and having persistent excretion of the virus, in order to offer optimal management of their infection, and strengthen prevention measures to avoid subsequent transmission of these selected variants.

## Data Availability

All data generated or analysed during this study are included in this published article.

## Abbreviations

COVID-19: CoronaVirus Disease 2019
SARS-CoV-2: Severe Acute Respiratory Syndrome Coronavirus 2
mAbs: Monoclonal antibodies
S: Spike
ICU: Intensive care unit
